# Beyond the Bench: Keeping Migrant Families Safe

**DOI:** 10.1289/ehp.112-a618

**Published:** 2004-08

**Authors:** Kimberly G. Thigpen

For the thousands of migrant farmworkers who come to the United States in search of jobs, this country may seem like the land of opportunity. For their children, however, it also offers the opportunity for serious injury or even death from exposure to household toxicants that parents may not be aware of or have the language ability to recognize as a hazard. A program of the Community Outreach and Education Program at the University of California, Davis, Center for Environmental Health Sciences is seeking to provide migrant families in Northern California with the skills to protect their children from accidental poisonings.

The Safety Literacy for Migrant Farm Worker Families: Childhood Poison Prevention project uses a variety of means to reach and educate migrant families. The centerpiece of the program is training conducted in Spanish at migrant housing centers, in which parents and staff are taught how to read and interpret safety warnings and emergency first aid instructions. These classes cover basic safety information on over-the-counter medicines and vitamins, household and personal care products (such as bleach, cleansers, and bug sprays), plants and other environmental toxicants, and pesticides used in the home or at work. Participants are also taught how to read labels, what to do in case of poisoning, and how to use 9-1-1 and poison control centers. Participants receive a variety of printed safety resources in Spanish such as booklets, posters, and stickers, and each trainee is given four safety latches to secure chemicals stored in their homes.

To date, classes have been provided at five Head Start/Early Head Start child development centers in Yolo County, and at a series of parenting sessions organized by Yolo Connections, an umbrella agency whose mission is to promote volunteerism, mentoring, and community partnership. The poison prevention training has also been presented at the annual California Office of Migrant Services conference to 83 staff members representing all 26 migrant housing centers in California.

Center staff have also developed safety materials for posting and distribution in target camps that illustrate the proper use and storage of toxic household substances, as well as emergency first aid techniques. The materials are distributed to Head Start, parenting classes, and migrant camps through collaboration with the California Poison Control Center, the California Office of Migrant Services, and the nonprofit California Human Development Corporation.

The Safety Literacy for Migrant Farm Worker Families program goes beyond education to preventive action through the distribution and installation of child-proof locks in migrant housing. By the 2004 harvest season, more than 1,000 safety latches will have been purchased by the project and installed in migrant camps throughout Yolo County. More information on the project can be found at the center’s website at **http://www.envtox.ucdavis.edu/cehs/**.

## Figures and Tables

**Figure:**
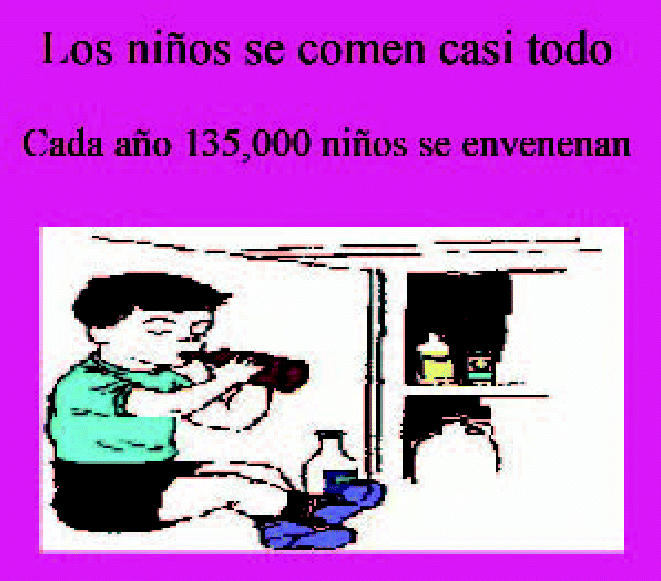
**Word to the wise.** A graphic reminds parents that “children will eat almost anything”!

